# Roles of ferroptosis in urologic malignancies

**DOI:** 10.1186/s12935-021-02264-5

**Published:** 2021-12-18

**Authors:** Shankun Zhao, Peng Li, Weizhou Wu, Qinzhang Wang, Biao Qian, Xin Li, Maolei Shen

**Affiliations:** 1 Department of Urology, Taizhou Central Hospital (Taizhou University Hospital), Taizhou, 318000 Zhejiang China; 2 Department of Urology, Qingdao Women and Children’s Hospital, Qingdao, 266000 Shandong China; 3 Department of Urology, Maoming People’s Hospital, Maoming, 525000 Guangdong China; 4 Department of Urology, The First Affiliated Hospital of Shihezi University Medical School, Shihezi, China; 5 Department of Urology, First Affiliated Hospital of Gannan Medical University, Ganzhou, 341000 Jiangxi China

**Keywords:** Ferroptosis, Regulated cell death, Urologic oncology, Mechanism, Therapy

## Abstract

Ferroptosis, an iron-dependent form of non-apoptotic cell death, is believed to strongly contribute to the pathogenesis of multiple cancers. Recently, the positive association between ferroptosis and urologic malignancies has drawn considerable attention, while a comprehensive review focused on this issue is absent. Based on this review, ferroptosis has been implicated in the development and therapeutic responses of prostate cancer, kidney cancer, and bladder cancer. Mechanistically, a large number of biomolecules and tumor-associated signaling pathways, including DECR1, PANX2, HSPB1, ACOT8, SUV39H1, NCOA4, PI3K-AKT-mTOR signaling, VHL/HIF-2α pathway, and Hippo/TAZ signaling pathway, have been reported to regulate ferroptosis in urologic cancers. Ferroptosis inducers, such as erastin, ART, CPNPs, and quinazolinyl-arylurea derivatives, exert potential therapeutic effects per se and/or enhance the anticancer response of other anticancer drugs in urologic oncology. A better understanding of ferroptosis may provide a promising way to treat therapy-resistant urologic cancers.

## Introduction

Urologic malignancies mainly include prostate cancer (PCa), bladder cancer, and kidney cancer. According to the GLOBOCAN 2020, there were approximately 2.4 million newly diagnosed cases of urologic oncologies in 2020, accounting for 12.5% of all cancer cases worldwide. Urologic malignancies were also responsible for a total of 767,208 deaths (7.7% of all cancer deaths) globally [1]. Among the three common urologic oncologies, PCa is the most prevalent cancer and the fifth leading cause of cancer-associated death among men worldwide [1]. As for bladder cancer, it is the second most common type of genitourinary cancer and ranks as the 10th most common malignancy worldwide in 2020 [1]. Although less common, about 431,288 cases of renal cancer were diagnosed in 2020, with 179,368 people dying from the disease worldwide that year. As the population ages, an increased incidence of urologic cancer is observed all around the world [1]. Radical surgical resection is the most important curative treatment for the early stage of urologic cancers. However, patients diagnosed in an advanced stage of disease face a high rate of morbidity and mortality, which constitutes a major challenge for the treatment and prognosis of urologic malignancies. In addition, the prognosis of patients with advanced-stage tumors remains abysmal due to resistance against chemo- and radiotherapy. As a result, the corresponding examinations and interventions of urologic cancers generate a significant financial burden.

There is growing evidence that an insight into the potential molecular mechanism of urologic oncologies may serve as a prerequisite to improve the therapies and develop a new treatment. There is a high need to find new clinical approaches to increase these tumors’ sensitivity to radiotherapy and chemotherapy in advanced urologic cancers. It is established that aberrant cell death contributes to the tumorigenesis of multiple malignant tumors, including urologic cancers. Recently, accumulating evidence demonstrates that ferroptosis, a novel iron-dependent form of cell death, plays a crucial role in various cancers, i.e., lung cancer [2], breast cancer [3], colon cancer [4], and glioblastoma [5]. Ferroptosis is driven by the declination of scavenging action of GPX4 on ROS and the iron-dependent accumulation of ROS [6]. Circulating iron exists in the form of Fe^3+^, which can be transported into the endosomes by transferrin receptor 1, where Fe^3+^ is subsequently reduced to Fe^2+^ [7]. From the endosomes, Fe^2+^ enters the cytosolic labile iron pool. Moreover, excess intracellular iron is stored in ferritin, which consists of both heavy and light chains [7]. This means that decreased iron storage and increased iron uptake may lead to iron overload in ferroptosis. Excess iron induces ferroptosis by generating excessive ROS via Fenton action. It is difficult to remove cellular ROS when the antioxidative capacity of the tumor cells drops significantly, leading to ferroptosis occurrence.

Ferroptosis also critically involves in the pathophysiological processes of urologic malignancies, which has attracted increasing attention from urologists and researchers. The objective of this review is to summarize all the current knowledge about ferroptosis in the tumorigenesis and progression of human urologic oncologies.

## Overview of ferroptosis

**Fig. 1:**
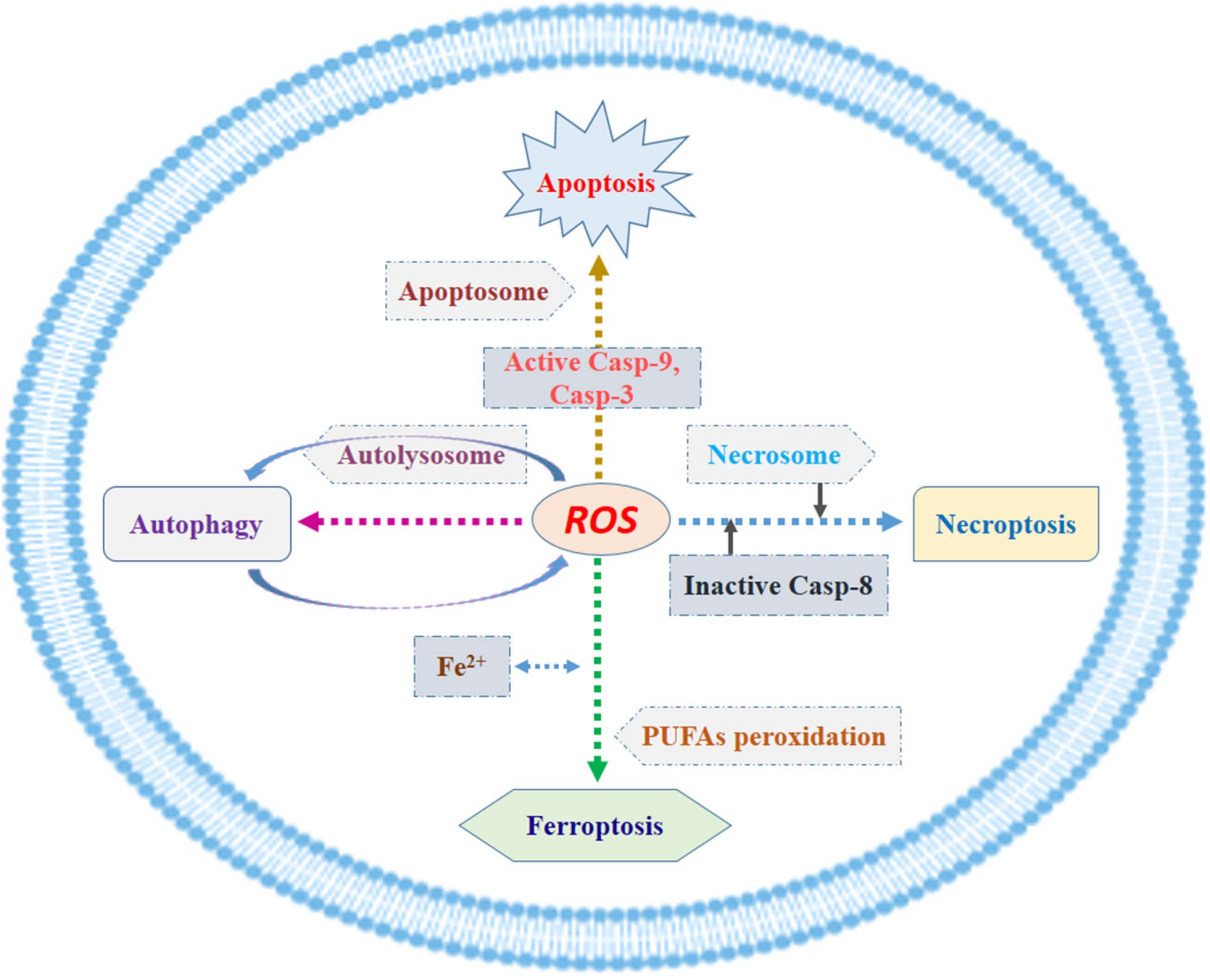
Roles of ROS in four common types of cell death. The level of cellular ROS lies at the core position of apoptosis, necrosis, autophagy, and ferroptosis, while apoptosome, necrosome, autolysosome, and Fe^2+^ plays a key role in the process of the four common types of cell death, respectively. *ROS* reactive oxygen species, *PUFA* polyunsaturated fatty acid, *Casp*- caspase

The inducer of ferroptosis first appeared in a 2003 study by Dolma et al. [8]. The authorsdiscovered a new anti-tumor drug that could induce cell death without causing nuclear morphology changes, DNA fragmentation, and caspase-3 activation, and subsequently found that caspase inhibitors could not reverse this process. Later, Yang et al. [9] demonstrated that RSL3 was involved in this novel cell death modality, and iron chelators could inhibit this mode of cell death. In 2012, Dixon et al. [10] observed that erastin increased the reactive oxygen species (ROS) level of the tumor cells, which induced tumor cell death. And interestingly, the addition of iron chelator significantly inhibited ROS accumulation and tumor cell death. According to this characteristic, this mode of cell death was identified as “ferroptosis”. Distinct from apoptosis, necrosis, and autophagy, ferroptosis is an oxidative iron-dependent form of cell death. However, as shown in Fig. 1, all these types of cell death are associated with the level of cellular ROS. Apoptosome, necrosome, autolysosome, and Fe^2+^ may play a key role in the process of apoptosis, necrosis, autophagy, and ferroptosis, respectively.

Ferroptosis differs from apoptosis and various forms of necrosis in many aspects. Morphologically, it is characterized by reduced mitochondrial volume, decreased mitochondrial cristae, and enhanced mitochondrial membrane density [11]. In term of gene regulation, ferroptosis can be regulated by ribosomal protein L8 (RPL8), iron-responsive element-binding protein 2 (IREB2), ATP synthase F0 complex subunit C3 (ATP5G3), citrate synthase, tetratricopeptide repeat domain 35 (TTC35), and acyl-CoA synthetase family member 2 (ACSF2) [12]. These genes maintain iron homeostasis by controlling ferric absorption, metabolism, and storage. Biochemically, ferroptosis is characterized by iron-dependent with the lethal accumulation of lipid peroxidation-induced cell death [12]. Ferroptosis can be triggered by the inactivation of glutathione peroxidase 4 (GPX4), which drives the hyper-peroxidation of lipids leading to cell death [13]. According to these characteristics listed above, ferroptosis is a new iron-dependent and non-apoptotic form of cell death.

## The roles of ferroptosis in cancers

Unlimited proliferative capacity is the major feature of cancer cells. Thus, induction of cell death may be an effective therapeutic target for cancers. Ferroptosis is a new type of tumor cell death, which plays an important role in multiple cancers [14]. PTPN18 is considered to serve as an oncogene in endometrial cancer, which promotes proliferation and metastasis of endometrial cancer cells [15]. Another study revealed that silencing of PTPN18 could induce ferroptosis by inhibiting GPX4 activity, leading to the suppression of endometrial cancer cell growth [16]. Apatinib, also known as Rivoceranib, is a tyrosine kinase inhibitor that selectively inhibits the vascular endothelial growth factor receptor-2 (VEGFR2). Zhao et al. [17] reported that apatinib could inhibit the proliferation of gastric cancer (GC) cells as well as the multi-drug-resistant GC cells by inducing ferroptosis through inhibiting GPX4 activity. Moreover, dysregulation of ferroptosis has also been found to be associated with the pathophysiological processes of colorectal cancer, breast cancer, and lung cancer, etc. [18–21].

Acquired resistance increases tumor aggressiveness, indicating chemotherapy failure for cancer therapy. Consequently, reversing chemoresistance is very important and particularly urgent in terms of cancer treatments. Erastin, a classic ferroptosis inducer, inhibits SLC7A11 (solute carrier family 7 member 11, also known as xCT) which mediates the cystine/glutamate antiporter activity in the system X^‾^c, thus significantly reduced the capability of cells to synthesize glutathione (GSH), leading to an increasing of lipid peroxidation and resulting in ferroptosis [22]. Erastin can also regulate ferroptosis via the voltage-dependent anion channel and p53 [23]. Erastin is believed to serve as a promising tool for cancer therapy due to its ability to enhance the sensitivity of chemotherapy and radiotherapy [23]. Cisplatin and docetaxel are the first-line chemotherapy drugs for multiple cancers treatment [24]. However, chemoresistance is still a major challenge to the treatment of multiple cancers. A recent study discovered that the combination of cisplatin and erastin could provide a greater antitumor activity than cisplatin alone in non-small cell lung cancer cells and colorectal cancer cells [25]. Similar to this finding, the co-delivery of erastin with docetaxel showed more effectiveness in decreasing cell viability and promoting cell death of resistant ovarian cancer cells than docetaxel alone [26]. Subsequently, a series of antineoplastic agents inducing ferroptosis of cancer cells have been developed, including sorafenib, bicalutamide, and artesunate [27–29]. According to the above promising findings, ferroptosis may provide an effective strategy for the treatment of resistant cancers.

Based on the current evidence, ferroptosis is commonly repressed in multiple cancers and functions as a tumor suppressor in cancer development. The activation of ferroptosis may kill specific cancer cells and suppress cancer growth, while inactivation of which, like inactivation of apoptosis, contributes to tumor development [30]. As reported, ferroptosis might serve as a p53-mediated activity during tumor suppression, while p53 mutation aggravated tumorigenesis by repressing the occurrence of ferroptosis [31]. In addition to p53, some other possible modulations/alterations in the ferroptosis pathway have also been recognized as the tumor suppressing mechanisms, including GPX4, SLC7A11, activating transcription factor 3 (ATF3), and BRCA1-associated protein 1 (BAP1), etc. [21].

Mounting studies have been conducted to better address the association between ferroptosis and urologic malignancies, while a comprehensive review about this issue is extremely scarce. In this study, we intended to summarize all the evidence on this topic to better facilitate the clinical understanding of this issue.

## Methods

We have conducted a systematic searching in four common databases [MEDLINE (PubMed), Cochrane Library, EMBASE (OVID), and PsychINFO] to identify the relevant studies prior to July 1, 2021. We only included those studies presented in English. The searching strategy used in the PubMed databases was: ((Ferroptosis) OR (Oxytosis)) AND (((((((((((((((((((("Prostatic Neoplasms"[Mesh]) OR (Prostate Neoplasms)) OR (Neoplasms, Prostate)) OR (Neoplasm, Prostate)) OR (Prostate Neoplasm)) OR (Neoplasms, Prostatic)) OR (Neoplasm, Prostatic)) OR (Prostatic Neoplasm)) OR (Prostate Cancer)) OR (Cancer, Prostate)) OR (Cancers, Prostate)) OR (Prostate Cancers)) OR (Cancer of the Prostate)) OR (Prostatic Cancer)) OR (Cancer, Prostatic)) OR (Cancers, Prostatic)) OR (Prostatic Cancers)) OR (Cancer of Prostate)) OR (((((((((((((((((("Urinary Bladder Neoplasms"[Mesh]) OR (Neoplasm, Urinary Bladder)) OR (Urinary Bladder Neoplasm)) OR (Neoplasms, Bladder)) OR (Bladder Neoplasms)) OR (Bladder Neoplasm)) OR (Neoplasm, Bladder)) OR (Bladder Tumors)) OR (Bladder Tumor)) OR (Tumor, Bladder)) OR (Tumors, Bladder)) OR (Urinary Bladder Cancer)) OR (Cancer, Urinary Bladder)) OR (Malignant Tumor of Urinary Bladder)) OR (Cancer of the Bladder)) OR (Bladder Cancer)) OR (Bladder Cancers)) OR (Cancer, Bladder))) OR (((((((((((((((((("Kidney Neoplasms"[Mesh]) OR (Kidney Neoplasm)) OR (Neoplasm, Kidney)) OR (Renal Neoplasms)) OR (Neoplasm, Renal)) OR (Neoplasms, Renal)) OR (Renal Neoplasm)) OR (Neoplasms, Kidney)) OR (Cancer of Kidney)) OR (Kidney Cancers)) OR (Renal Cancer)) OR (Cancer, Renal)) OR (Cancers, Renal)) OR (Renal Cancers)) OR (Cancer of the Kidney)) OR (Kidney Cancer)) OR (Cancer, Kidney)) OR (Cancers, Kidney))). We also identified the additional studies by manual inspection of reference lists in the related articles.

**Fig. 2:**
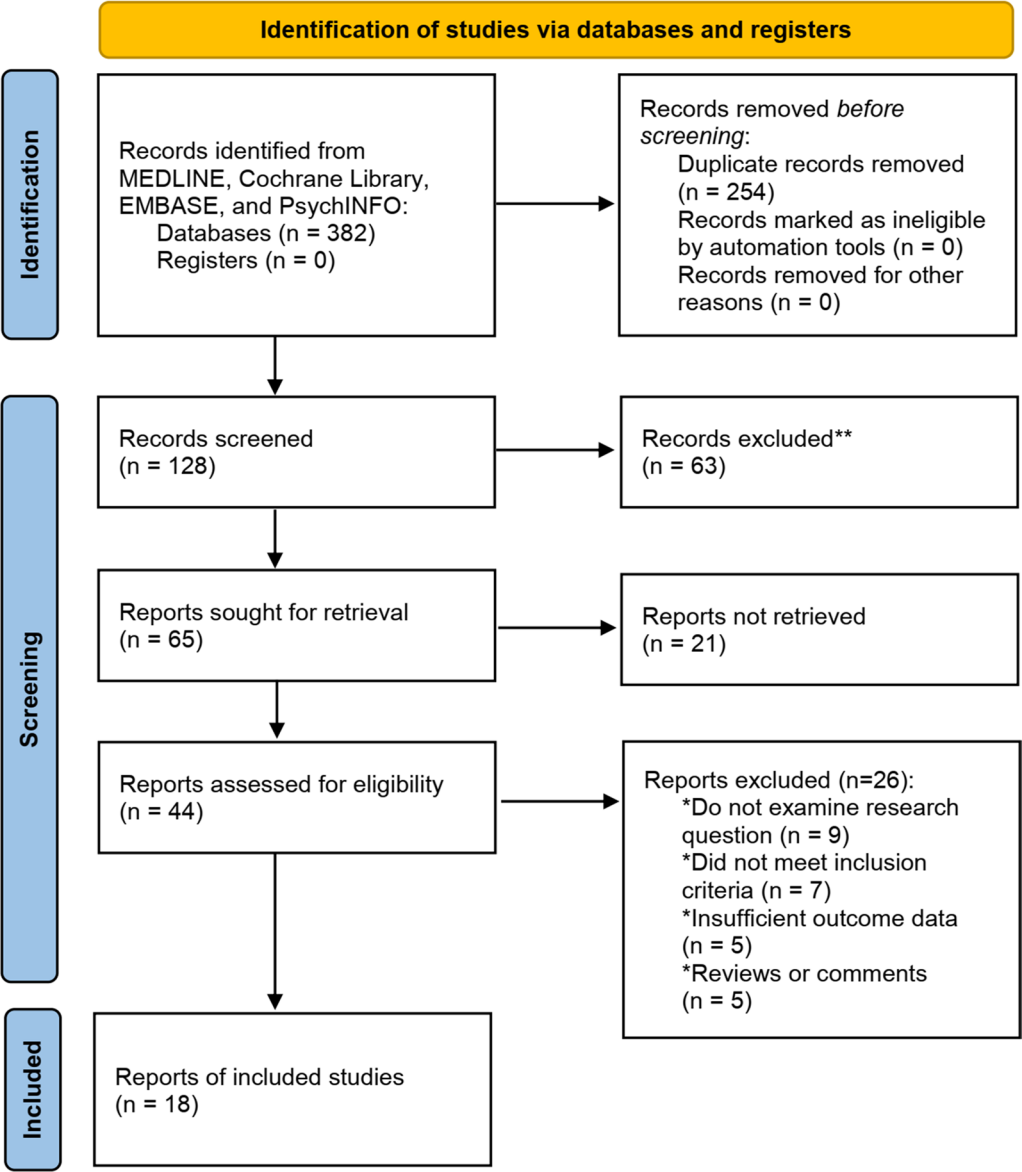
Flow chart of study selection to identify the relevant studies reported with the association between ferroptosis and urologic malignancies

Figure 2 showed the searching flowchart for identifying the eligible studies reporting the association between ferroptosis and urologic malignancies. In the initial database search, 432 publications were detected, of which 127 came from MEDLINE, 101 from EMBASE, 81 from the Cochrane Library, and 73 from the PsychINFO database. After excluding duplicates and those studies with reasons, 18 eligible studies [28,38,41,50,65,67,70,73,79,86,89,96,101,111,118,119,121,122] were finally included, of which 8 studies reported with PCa, 8 for kidney cancers, and 2 for bladder cancers. The biomolecular mechanisms of ferroptosis in urologic malignancie mentioned in the 18 included studies are summarized in Table 1.

**Table 1:** Mechanisms of ferroptosis in urological cancers

Cancer type	Biomolecular mechanism	References
Prostate cancer	HDECR1 silencing induced ferroptosis by accumulation of PUFAs and then promoting ROS generation	[38]
	Silencing of PANX2 promoted ferroptosis by suppressing the expression of Nrf2	[41]
	PI3K/AKT/mTOR inhibited ferroptosis via upregulating SREBP1/SCD1	[50]
	HSPB1inhibited ferroptosis by promoting iron uptake and increasing lipid ROS production	[65]
	Bicalutamide-iron combination induced ferroptosis	[28]
	The combination of erastin and docetaxel induced ferroptosis	[111]
	CHAC1 inhibits cell viability and increases the sensitivity of prostate cancer cells to docetaxel by inducing ferroptosis	[67]
	Therapy-induced lipid uptake and remodeling underpin GPX4 dependence and ferroptosis hypersensitivity	[70]
Kidney cancer	VHL/HIF-2α induced ferroptosis via elevating lipid peroxidation levels through HILPDA	[73]
	TAZ silencing reduce sensitivity to erastin-induced ferroptosis by downregulating the expression of EMP1-NOX4	[79]
	ACOT8 inhibited ferroptosis	[86]
	Repression of SUV39H1 induced ferroptosis via enhancing DPP4 activity	[89]
	The low-expression of NCOA4 conferred ccRCC cells resistance to ferroptosis by increasing FTH and FTMT expression levels	[96]
	GPX1 induced ferroptosis in KIPP	[101]
	ART induced ferroptosis and enhanced the anti-tumor effect of sunitinib	[118, 119]
Bladder cancer	CPNPs targeted to EDNRB via EDN3-CPNPs and thereby induced ferroptosis	[121]
	Quinazolinyl-arylurea derivatives induced ferroptosis through ROS generation and GSH depletion	[122]

## Potential role of ferroptosis in PCa

### DECR1 inhibits ferroptosis in PCa

Lipid metabolism is closely related to the occurrence, development, and progression of various cancers, including PCa [32, 33]. Increasing dietary polyunsaturated fatty acid (PUFA) may reduce the risk of PCa by affecting oxidative stress and tumor apoptosis [34, 35]. 2,4-Dienoyl-CoA reductase 1 (DECR1), a rate-limiting enzyme in PUFA oxidation, is significantly upregulated in prostate tumor samples and mouse models of castration resistance but downregulated in breast cancer [36, 37]. This demonstrates that DECR1 plays a diversified role in different types of cancers. A recent study reported that silencing of DECR1 significantly inhibited the proliferation of PCa cells and the growth of castration-resistance PCa (CRPC) in vivo [38]. In addition, DECR1 knockdown caused accumulation of polyunsaturated fatty acids (PUFAs), increasing the susceptibility to lipid peroxidation, and triggered ferroptosis [38]. Intriguingly, DECR1 knockdown elevates the expression of the lipid-detoxifying enzyme glutathione peroxidase 4 and sensitizes CRPC cells to ferroptosis [36]. Ferroptosis can be repressed by the inhibitors of ferroptosis (i.e., liproxstatin or Trolox), regaining the proliferative capacity of the cancer cells [36]. Moreover, high DECR1 expression is related to shorter disease-free survival in patients with PCa, which is of great importance for predicting the prognosis [38]. The above evidence elucidates the association between DECR1 and ferroptosis in PCa.

### PANX2 inhibits ferroptosis thus facilitating PCa progression

PANX2, a member of the pannexin family, and pannexin 1 are abundantly expressed in the central nervous system and are coexpressed in various neuronal populations (https://www.proteinatlas.org/ENSG00000073150-PANX2). Multiple transcript variants encoding different isoforms have been found for PANX2. Besides, this gene was also considered to play an essential role in multiple cancers. Recent studies have shown that PANX2 was dramatically upregulated in cholangiocarcinoma and renal cell carcinoma [39, 40]. However, it is unclear how to affect the development of PCa. A new study demonstrated that PANX2 expression levels are significantly upregulated in PCa tissues and cell lines as well as being correlated with Gleason score [41]. Therefore, PANX2 may be a useful predictor for the severity in PCa patients. In addition, PANX2 silencing remarkably inhibited the proliferation, migration, and invasion capacities of the PCa cells [41]. Moreover, PANX2 knockdown substantially reduces the nuclear factor erythroid 2-related factor 2 (Nrf2) and its downstream gene expressions [41]. Nrf2, a major regulator of the antioxidant response, plays a critical role in mitigating ferroptosis by regulating SLC7A11 [42, 43]. Sun et al. [44] found that activation of Nrf2 promoted the development and progression of hepatocellular carcinoma cells via protecting against ferroptosis. Another study developed by Liao et al. [41] indicated that silencing of PANX2 significantly suppressed PCa progression, and speculated the underlying mechanism might be correlated to PANX2-knockdown that promoted ferroptosis by inhibiting the expression of Nrf2. Strikingly, oltipraz, an essential activator of Nrf2, can reverse the inhibitory effects of PANX2 silencing on PCa cell proliferation, metastasis, invasion, and ferroptosis [41]. However, in contrast, PANX2 decreases cell/tumor growth in C6 glioma, indicating PANX2 also plays the role of a cancer suppressor in some types of cancers [45].

### PI3K/AKT/mTOR signaling suppresses ferroptosis via SREBP1/SCD1

Alterations in the phosphatidylinositol 3-kinase-serine/threonine kinase AKT (PI3K-AKT) signaling pathway are associated with the advancement of multiple cancers, including PCa. According to statistics, 70% of advanced PCa exhibits activation of the PI3K/AKT pathway [46]. mTOR, a downstream player of the PI3K/AKT signaling pathway, regulates tumor cellular functions. It has been reported that high cholesteryl ester levels played an important role in PCa progression [47]. Yue et al. [48] demonstrated that upregulation of the PI3K/AKT/mTOR pathway could induce the accumulation of cholesteryl ester, thus facilitating PCa progression. A recent study showed that PI3K/AKT/mTOR pathway participated in the progression of non-small-cell lung cancer by regulating ferroptosis [49]. Yi et al. [50] reported that the PI3K-AKT-mTOR pathway inhibited ferroptosis, while inhibition of PI3K and mTOR could activate ferroptosis in cancer cells. Also, the authors suggested that the PI3K-AKT-mTOR pathway might serve as a clinical marker for specific types of cancers [51]. Presently, the association between PI3K/AKT/mTOR pathway-mediated ferroptosis and the development of PCa is still under investigation. A new study discovered that PC-3 cells line carrying PIK3CA activating mutation appeared to be more resistant to RSL3-induced ferroptosis [50]. Furthermore, pharmacological inhibition of the PI3K/AKT/mTOR pathway could sensitize PC-3 cells to ferroptosis induction [50]. Nrf2 and sterol regulatory element-binding proteins 1 (SREBP1) are the main regulator in lipid metabolism, which can be both regulated by this pathway [52, 53]. NRF2-knockout had no measurable effect on ferroptosis induced by RSL3, but SREBF1 silencing sensitized ferroptosis and lipid peroxidation in PC-3 cells and decreased the expression of stearoyl-coenzyme A desaturase 1 (SCD1) [50]. SCD1 is an important enzyme converting saturated fatty acids to monounsaturated fatty acids, and then inhibiting ferroptosis. It was reported that SCD1 exerted a suppressive effect on ferroptosis in lung cancer and gastric cancer [54, 55]. Further study found that in these SREBF1-knockout cells, pharmacological inhibition of this pathway failed to further increase RSL3-induced ferroptosis [50]. Mechanically, activation of the PI3K/AKT/mTOR pathway promotes the development of PCa through SREBP1/SCD1-mediated ferroptosis [50]. Importantly, in a PCa xenograft mouse model with activation of PI3K-AKT-mTOR pathway, imidazole ketone erastin, a ferroptosis induction agent, alone had no effects on tumor growth, but its combination with Temsirolimus, a mTORC1 inhibitor, resulted in dramatic tumor regression [50]. The above studies may supply a novel therapeutic approach for the treatment of PCa by harboring an activating mutation of the PI3K-AKT-mTORC1 pathway.

### HSPB1 negatively regulate ferroptosis

HSPB1 (heat shock protein family B member 1, also known as mouse HSP25 or human HSP27), a small heat-shock protein, exerts anti-apoptotic or anti-cell death effects by degrading unfold or misfold proteins [56]. Moreover, the phosphorylation of HSPB1 could significantly enhance its biological effect [57]. As reported, HSPB1 was highly expressed in various tumors, including PCa [58–60]. A previous study showed that HSPB1 significantly decreased iron intracellular levels in fibroblasts of the heart [61–63]. However, its role in ferroptosis is unknown. A recent study showed that erastin not only induced ferroptosis but also upregulated the expression of HSPB1 [64]. Sun et al. [65] demonstrated that HSPB1 knockdown dramatically increased erastin-induced ferroptosis in PCa cell lines. The author also found that erastin significantly inhibited tumor growth in the xenograft model of PCas [65]. Importantly, HSPB1 knockdown resulted in a higher tumor inhibition effect [65]. Furthermore, protein kinase C (PKC) inhibitors, by blocking HSPB1 phosphorylation, significantly increased growth inhibition following erastin treatment by promoting iron uptake and increasing lipid ROS production [65]. The above studies indicate that HSPB1 or HSPB1 phosphorylation functions as a negative regulator of ferroptosis in PCa.

### CHAC1 inhibits cell viability by inducing ferroptosis

ChaC glutathione specific γ‑glutamylcyclotransferase 1 (CHAC1), one of the components of the unfolded protein response (UPR) pathway, can be induced in response to endoplasmic reticulum stress [66]. Recently, He et al. [67] discovered that CHAC1 expression levels were correlated with PCa cell viability and the GSH levels. The expression of CHAC1 was significantly upregulated when treated with a ferroptosis activator. In addition, overexpression of CHAC1 resulted in significant incrementing of the intracellular lipid peroxides levels and the declination of the GPX4 levels. Mechanically, He et al. found that CHAC1 inhibited the viability of PCa cells and elevated their sensitivity to docetaxel by inducing endoplasmic reticulum stress and ferroptosis. And this inhibition could be eliminated by adding a ferroptosis inhibitor [67].

### GPX4 dependence and ferroptosis hypersensitivity of persister cells

GPX4, a selenocysteine enzyme, is important for protection against lipid peroxidation injury and the induction of ferroptosis. It was reported that GPX4 dependence and ferroptosis hypersensitivity could be detected in multiple malignancies under different therapies, which was considered to be associated with acquired therapy resistance and tumor relapse [68, 69]. Tousignant, along with their colleagues [70], found that GPX4 dependence and ferroptosis hypersensitivity of persister cells induced by androgen-targeted therapies were correlated to enhanced lipid uptake and PUFA enrichment of membrane lipids. The authors also reported that therapy-induced lipid remodeling and lipid supply plasticity were the key mechanisms that enhanced ferroptosis hypersensitivity in persister PCa cells.

**Fig. 3:**
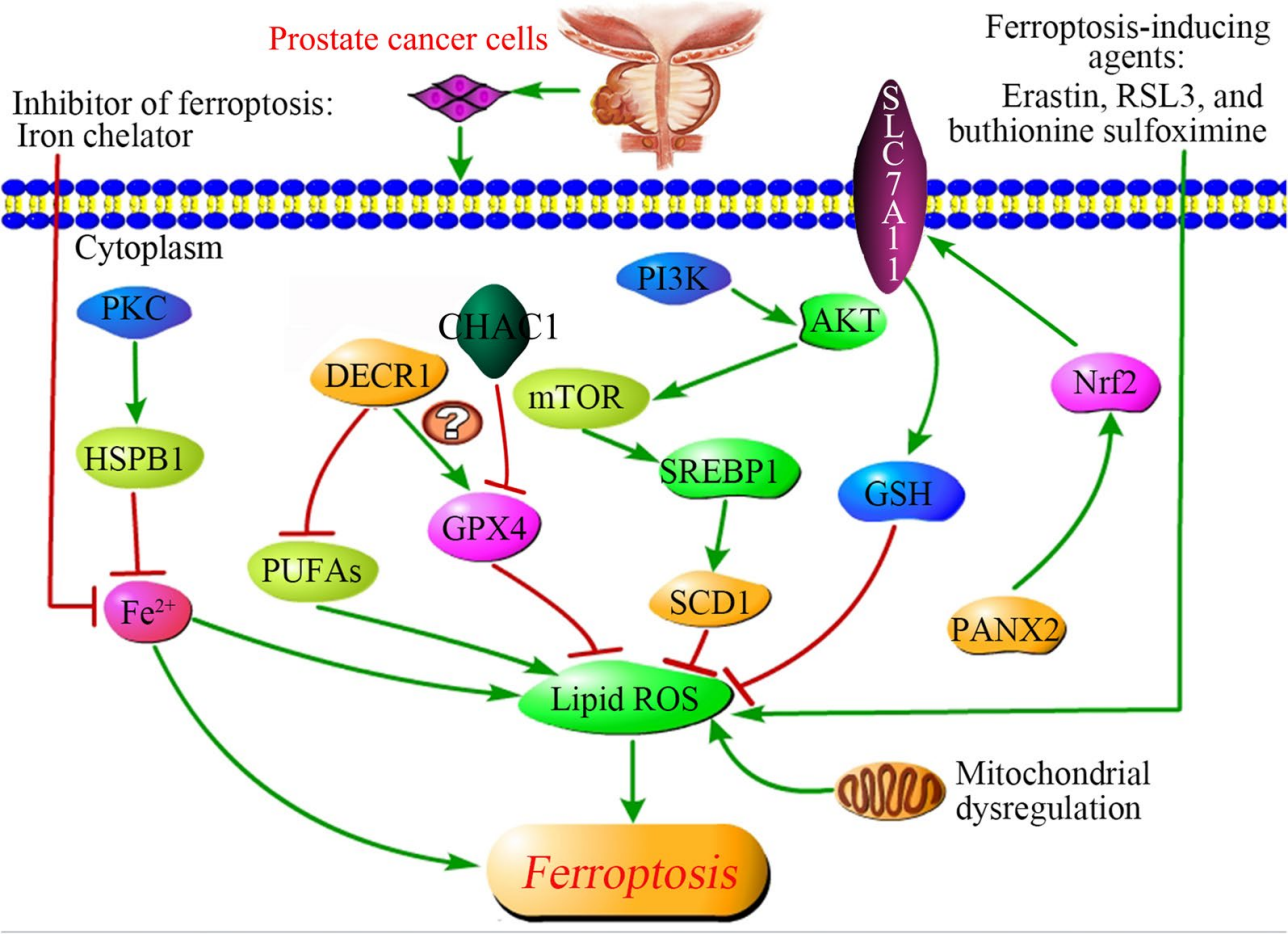
Signaling pathways regulating ferroptosis in prostate cancer. PKC represses Fe^2+^ uptake by promoting HSPB1 phosphorylation, thus decreasing lipid ROS production. DECR1 is negatively associated with PUFAs, but positively associated with the expression of GPX4, affecting ferroptosis through the ROS. CHAC1 increases the intracellular lipid peroxides levels by inhibiting the GPX4 levels. SREBP1/SCD1, which are promoted by the PI3K/AKT/mTOR pathway, contributes to the suppression of ROS, resulting in mitigating ferroptosis. SLC7A11, a cystine/glutamate antiporter that imports cystine into cells while exporting glutamate, decreases lipid ROS by enhancing GSH levels. SLC7A11 can be also upregulated by PANX2 that mediated by Nrf2, a major regulator of the antioxidant response. Mitochondrial dysregulation plays a critical role in promoting ferroptosis. Iron chelator inhibits ferroptosis by suppressing Fe^2+^, while erastin, RSL3, and buthionine sulfoximine induces ferroptosis by enhancing lipid ROS. ROS locates on the central position of multiple ferroptosis-related genes. *ROS* reactive oxygen species, *PKC* protein kinase C, *HSPB1* heat shock protein family B member 1, *DECR1* 2,4-dienoyl-CoA reductase 1, *DECR1* ChaC glutathione specific γ‑glutamylcyclotransferase 1, *PUFAs* polyunsaturated fatty acid, *GPX4* glutathione peroxidase 4, *PI3K-AKT* phosphatidylinositol 3-kinase-serine/threonine kinase AKT, *SREBP1* sterol regulatory element-binding proteins 1, *SCD1* stearoyl-coenzyme A desaturase 1, *GSH* glutathione, *Nrf2* nuclear factor erythroid 2-related factor 2, *SLC7A11* solute carrier family 7 member 11

Figure 3 displays the signaling pathways regulating ferroptosis in PCa.

## Regulation of ferroptosis in renal carcinoma

### VHL/HIF-2α pathway regulate ferroptosis by HILPDA in ccRCC

Clear cell renal cell carcinoma (ccRCC) is the most frequent type of renal cell carcinoma (RCC) and is closely associated with mutations of the von Hippel-Lindau (VHL) gene [71]. Its inactivation increase HIF-2α, a hypoxia-inducible factor, levels in ccRCC tumors. According to a number of studies, HIF-2α could regulate lipid metabolism and play a pro-tumorigenic role in the development of ccRCC [72]. However, the role of the VHL/HIF-2α pathway on ferroptosis remains unclear in ccRCC. Miess et al. [73] reported that restoration of pVHL function significantly inhibited tumor formation and decreased the expression of HIF-2α in ccRCC cells. Another study from Zou et al. [74] demonstrated that HIF-2α selectively enriched PUFAs by activating the expression of hypoxia-inducible and hypoxia inducible lipid droplet associated protein (HILPDA). It has also been reported that VHL reconstitution could reduce the sensitivity of ccRCC cells towards oxidative stress and inhibit ferroptosis [73]. In addition, the numbers and size of lipid droplets were strongly increased, which drove ferroptosis by lipid peroxidation in VHL-deficient renal cancer cells [73]. Mechanistically, VHL/HIF-2α pathway might induce ferroptosis by elevating lipid peroxidation levels via HILPDA. It is consistent with a previously published study revealing that pheochromocytoma and paraganglioma, displaying mutations in the VHL/HIF pathway, also exhibited aberrant cytoplasmic lipid levels [75]. Collectively, VHL/HIF-2α pathway-related ferroptosis may be a potential onco-target for the treatment of ccRCC.

### The Hippo effectors TAZ regulates ferroptosis via affecting EMP1-NOX4 in RCC

The Hippo signaling pathway is a highly conserved kinase cascade and plays a key role in multiple cancers by activating downstream effectors (YAP and TAZ) [76]. The cell density not only regulated this pathway but also had an important effect on ferroptosis sensitivity [77]. Yang et al. [77] reported that TAZ silencing rendered ovarian cancer cells resistant to ferroptosis, while TAZ overexpression sensitized cells to ferroptosis. However, whether TAZ regulates ferroptosis in RCC is also unclear. Recently, Ruan et al. [78] demonstrated that high TAZ expression in renal cancer was positively correlated with poor prognosis and distant metastasis. Another study suggested that TAZ knockdown in the low-density RCC cells significantly reduced the sensitivity to erastin-induced ferroptosis and down-regulated the expression of the epithelial membrane protein 1 (EMP1) [79]. Importantly, EMP1 overexpression reverse TAZ knockdown-induced ferroptosis resistance [79]. In addition, EMP1 silencing also conferred ferroptosis resistance in RCC cells and decreased the expression level of NOX4, an important regulator of lipid peroxidation for ferroptosis [79]. It has also been found that nicotinamide adenine dinucleotide phosphate oxidase 4 (NOX4) inhibitor protected RCC cells from ferroptosis and NOX4 overexpression increased the sensitivity of RCC cells to erastin treatment [79]. The above studies indicated that TAZ might regulate cell density-regulated ferroptosis by affecting EMP1-NOX4, which was in line with a previous study [80]. Therefore, TAZ showed a promising therapeutic potential for RCC. Whether the downstream effectors of the Hippo/YAP signaling pathway participate in the regulation of ferroptosis needs to be further determined.

### ACOT8 inhibits ferroptosis thus promoting ccRCC progression

Acyl-CoA thioesterase 8 (ACOT8), a member of the acyl-CoA thioesterase superfamily, catalyzes the hydrolysis reaction of fatty acyl-CoA ester and releases coenzyme A and free fatty acid (FFA), which implies its essential role in regulating lipid metabolism for fatty acids [81]. It has been reported that ACOT8 participates in multiple cancer tumorigeneses, such as hepatocellular carcinoma, colorectal cancer, and lung adenocarcinoma. The reprogramming of oxidative phosphorylation (OXPHOS) and fatty acid metabolism might play pivotal roles in the development of ccRCC [82]. However, it is unclear whether ACOT8 can modulate the ccRCC tumorigenesis [83–85]. Recently, Xu et al. [86] found that the expression level of ACOT8 was significantly downregulated in ccRCC samples, but patients with more advanced TNM stages had a tendency to express higher ACOT8. Meanwhile, higher ACOT8 expression was significantly correlated to poor prognosis of ccRCC and involved in fatty acid metabolism [86]. Similar to this finding, the expression of ACOT8 was increased in hepatocellular carcinoma and lung adenocarcinoma, indicating it might serve as a potential prognostic biomarker of these cancers [84, 85]. Based on the above evidence, ACOT8 may exert discordant actions in ccRCC tumorigenesis and progression. Additionally, the expression of ACOT8 was positively associated with ferroptosis-suppressing GPX4, but was negatively associated with ferroptosis-promoting TAZ, HILPDA, and HIF-2α [86]. It has also been found that oxidative phosphorylation (OXPHOS) expression was reduced at the stage of pathogenesis and increased during progression [86]. Additional to its effects on fatty acid metabolism and OXPHOS, ACOT8 may also affect ferroptosis, thus participating in ccRCC development and progression. At present, the specific molecular mechanisms for ACOT8 regulation of ferroptosis in ccRCC remains to be further studied.

### Repression of SUV39H1 induces ferroptosis through the upregulation of DPP4 expression in ccRCC

Suppressor of variegation 3–9 homolog 1 (SUV39H1), the first characterized mammalian domain-containing histone methyltransferase, catalyzes tri-methylation of histone 3 lysine 9 (H3K9me3) [87]. It is generally reported to be a tumor suppressor. However, increasing evidence shows that SUV39H1 may also have oncogenic properties. SUV39H1 has been reported to facilitate tumor progression by inhibiting the expression of retinoblastoma [88]. Few studies have investigated the role of SUV39H1 in ccRCC. A recent study showed that silencing of SUV39H1 markedly inhibited ccRCC cell proliferation and tumor growth in vivo and in vitro via inducing G2/M phase cell cycle arrest [89]. Furthermore, the intracellular lipid ROS, intracellular iron, and Fe^2+^ level were significantly increased in ccRCC cells with SUV39H1 knockdown, and ferroptosis was observed [89]. Dipeptidyl-peptidase-4 (DPP4), a transmembrane glycoprotein, could induce ferroptosis by binding to NADPH oxidase 1 (NOX1) and functioning in promoting lipid oxidation [90]. DPP4 also plays an important role in multiple cancers, including ccRCC [91, 92]. Wang et al. [89] reported that SUV39H1 knockdown significantly upregulated the expression of DPP4 via modulation of H3K9me3, the DPP4 promoter. Additionally, DPP4 knockdown partially reversed cell growth in SUV39H1-silenced ccRCC cells [89]. Importantly, ccRCC patients with low DPP4 transcript levels had a shorter median overall survival (38.5 months) compared with the high DPP4 expression group (42.3 months) [89]. Accordingly, SUV39H1 knockdown may modulate the expression of H3K9me3 that contributes to ferroptosis.

### NCOA4 contributes to ferroptosis by targeting FTH and FTMT in ccRCC

The nuclear receptor coactivator 4 (NCOA4) is a well-known regulator of ferritinophagy [93]. Its overexpression promotes ferritinophagy and drives ferroptosis, which regulates the occurrence of various human diseases. FitzGerald et al. [94] reported that NCOA4 overexpression was associated with a 15% reduction in PCa risk. However, the associations between NCOA4 and kidney cancer also remain unclear. A recent study revealed that NCOA4 expression decreased in ccRCC tumor tissue compared to normal tissue [95]. In addition, low NCOA4 expression in ccRCC cases was correlated with shorter overall survival [95]. Mechanistically, low-expression NCOA4 might increase ferritin heavy chain (FTH) and ferritin mitochondrial (FTMT) expression, two iron storage proteins, thereby protecting ccRCC cells from ferroptosis [96].

### GPX1 induces ferroptosis in KIPP

Glutathione peroxidase-1 (GPX1) is an important member of the GPX family as GPX4, which plays a role in cancer cell proliferation invasion, metastasis, and apoptosis [97]. GPX1 is considered to serve as an oncogene or tumor suppressor in different cancers [98]. Meng et al. [99] demonstrated that GPX1 silencing induced epithelial–mesenchymal transition (EMT) and inhibited the growth of pancreatic cancer cell lines. However, a study from Guerriero et al. [100] indicated that high expression of GPX1 was associated with poor prognosis in hepatocellular carcinoma. A more recent study showed that high GPX1 expression in kidney renal papillary cell carcinoma (KIPP) patients had a better prognosis than those with lower expression [101]. Further investigation indicated that GPX1 might be involved in ferroptosis pathways via interaction with GPX4 [101]. Additionally, GPX1 also participated in ROS metabolic process and leukocyte activation involved in immune responses [101]. The mechanisms of how GPX1 exerts its tumor suppression effect need further research in KIPP.

**Fig. 4:**
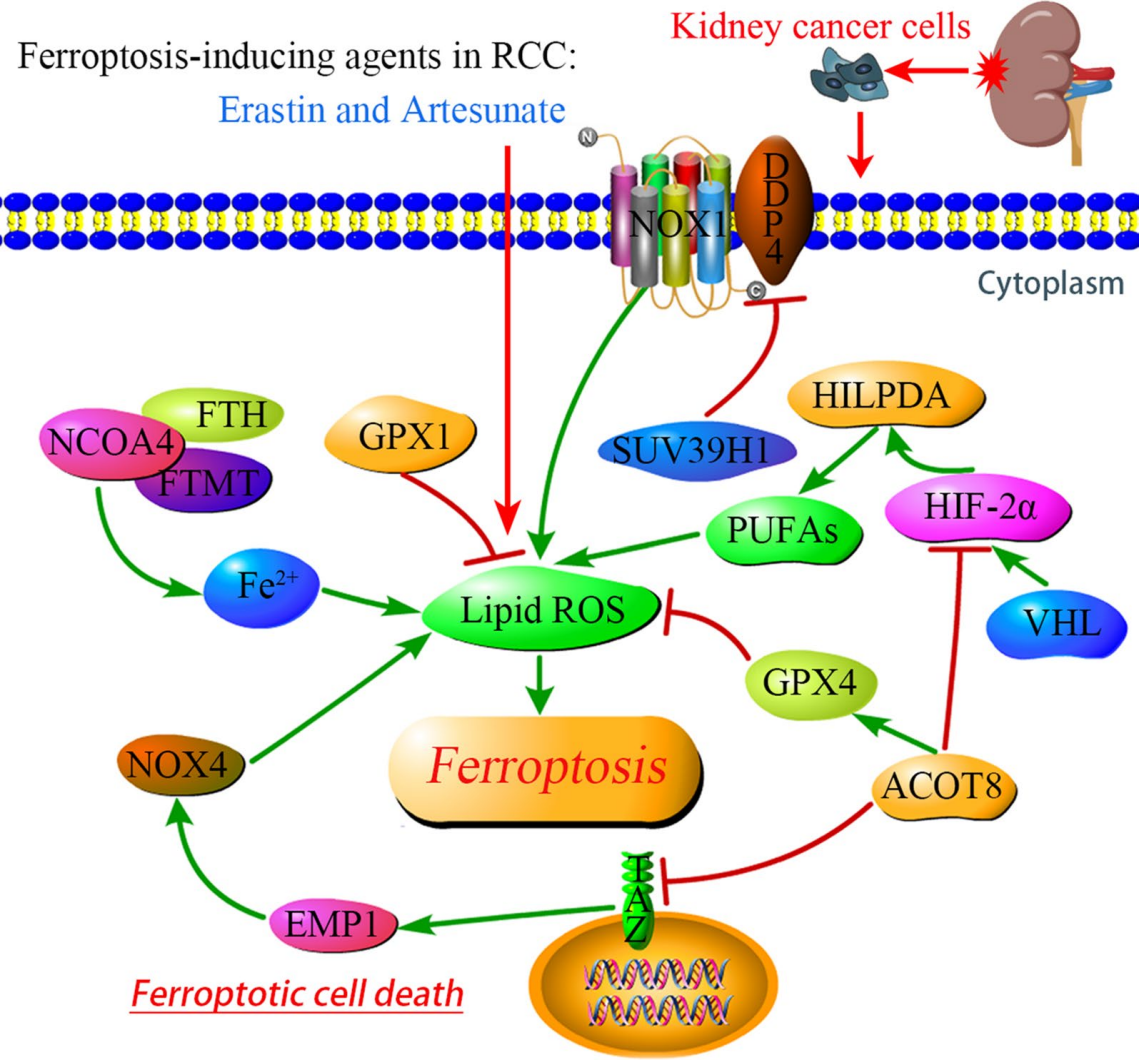
Signaling pathways regulating ferroptosis in RCC. DPP4 induces ferroptosis by binding to NOX1 and functioning in promoting ROS, while SUV39H1 downregulates the expression of DPP4. NCOA4 targeting FTH and FTMT thus increase the ferrous iron (Fe^2+^) and ROS. The Hippo effector TAZ enhances ROS via the EMP1-NOX4 signal axis. ACOT8 positively regulates GPX4 expression, but is negatively associated with TAZ and HIF-2α. HIF-2α upregulates by the VHL, and is positively associated with HILPDA to activate the expression of PUFAs, thus strengthens the accumulation of ROS. GPX1 participates in ROS metabolic process that suppressing ROS directly. In RCC, ferroptosis-inducing agents erastin and artesunate elevate the lipid ROS, thus promote ferroptotic cell death. *NCOA4* nuclear receptor coactivator 4, *FTH* ferritin heavy chain, *FTMT* ferritin mitochondrial, *GPX1* glutathione peroxidase-1, *SUV39H1* suppressor of variegation 3–9 homolog 1, *DPP4* dipeptidyl-peptidase-4, *ACOT8* acyl-CoA thioesterase 8, *VHL* von Hippel-Lindau, *HIF-2α* a hypoxia-inducible factor, *HILPDA* hypoxia inducible lipid droplet associated protein, *NOX4* nicotinamide adenine dinucleotide phosphate oxidase 4, *EMP1* epithelial membrane protein 1, *TAZ* transcriptional coactivator with PDZ-binding motif

Figure 4 displays the signaling pathways regulating ferroptosis in RCC.

## The impact of ferroptosis on bladder cancer

There may be a close association between downregulation or suppression ferroptosis and the proliferation of bladder cancer cells. A previous study reported that a low free iron concentration significantly favored the proliferation of bladder cancer cells [102]. In contrast, when Gallium is bound to transferrin, the level of free iron in bladder cancer cells was increased, thus inhibiting the proliferation [102]. Mazdak et al. [103] examined the serum iron expression levels in 51 patients with bladder cancer and 58 controls and found that patients with bladder cancer have lower levels of serum iron compared to healthy controls. The above evidence suggests that an increased level of free iron and serum iron may be involved in bladder cancer tumorigenesis and further prospective studies are needed.

Currently, there are five publications [104–108] have reported the association between ferroptosis and bladder cancer through a bioinformatics analysis. Yan et al. [104] identified a 6-gene signature based on the potential prognostic ferroptotic regulatory genes, and found that three genes (CRYAB, SQLE, and ZEB1) were positively associated with clinical stage of bladder cancer. Yang et al. [105] have discovered a novel prognostic model in bladder cancer integrating nine ferroptosis-related differentially expressed genes, including ALB, BID, FADS2, FANCD2, IFNG, MIOX, PLIN4, SCD, and SLC2A3, which could be applied for prognostic prediction in bladder cancer patients. Luan et al. [106] have identified four ferroptosis-associated genes in bladder urothelial carcinoma (CRYAB, TFRC, SQLE and G6PD) by conducting a bioinformatics analysis, which may accurately predict prognosis in bladder cancer patients. Liang et al. [107] constructed a new prognostic model with seven ferroptosis-related genes, including NOX1, GCLM, ACSL4, ALOX5, ACACA, ZEB1, and FADS2. Targeting these genes could be used for the prognosis prediction and treatment option for bladder cancer. Besides, several ferroptosis-related long noncoding RNAs (lncRNAs) and their nomograms have also been identified to predict the overall survival of bladder cancer, i.e., LINC00942, MAFG-DT, AL049840.3, AL136084.3, and OCIAD1-AS1, etc. [108].

## The roles of ferroptosis in the treatment of PCa and RCC

### Ferroptosis inducers enhance the efficacy of endocrine therapy and chemotherapy for PCa

At present, there is no effective therapy for advanced PCa. Androgen deprivation therapy (ADT) and chemotherapy are still the basic treatment for PCa. However, drug resistance is one of the major barriers to pharmacological therapy for PCa. It has been shown that ferroptosis boosts the cell death of cancer cells and plays a crucial role in the management of multiple cancers, including PCa [109]. Bordini et al. [28] suggested that iron at high concentration was toxic to all PCa cell lines, reducing cell proliferation and tumor growth compared with untreated cells in vitro and in vivo. In PC-3 xenografts, bicalutamide-iron combination successfully suppressed xenograft tumor growth, while single compounds were ineffective [28]. This might be due to the promoting effect of bicalutamide on aldoketo reductases, which exacerbating iron-induced oxidative damage [28]. Erastin and RSL3, the recognized ferroptosis-inducing agent, play a key role in the management of multiple cancers. Ghoochani et al. [110] found that treatment-resistant PCa cells growth and migration were significantly decreased and the tumor growth of treatment-resistant PCa was significantly delayed by erastin or RSL3. In addition, Yang et al. [111] reported that an erastin-docetaxel combination enhanced the tumor growth inhibition efficacy of docetaxel on castration-resistant PCa, which might be due to the suppressive expression of both the full-length and splice variants in the cancer cells. Similar to the above research, isothiocyanate (ITC)-containing hybrid AR antagonist (ITC-ARi) combined with GSH synthesis inhibitor buthionine sulfoximine (BOS) could induce ferroptosis and efficiently decrease the activity of AR, significantly enhancing anti-CRPC activities of ITC-ARi. The drug combination that caused cell viability was effectively rescued by iron chelator, an inhibitor of ferroptosis [112]. The combination of the two drugs mentioned above might increase the accessibility of ITC-ARi and elevated the free intracellular ferrous iron [112]. These results demonstrate that ferroptosis inducers may possess a potential efficacy in the treatment of CRPC and advanced PCa.

### Synergistic antitumor activity of artesunate and sunitinib through ferroptosis in RCC cells

RCC is a hypervascular cancer that can be controlled by tyrosine kinase inhibitor (TKI) sunitinib, an angiogenesis inhibitor [113]. However, therapy resistance is still a challenge in RCC therapy. Therefore, it is urgent to search for complementary therapeutic approaches to increase its effectiveness. Artesunate (ART) is a semi-synthetic derivative of artemisinin and has better anti-tumor activity than artemisinin via reducing the expression of angiogenic proteins [114, 115]. Previous studies indicated that ART synergized with TKI sorafenib to induce ferroptosis in hepatocellular carcinoma and enhanced the anti-tumor effect of the TKI sorafenib [116, 117]. A recent study demonstrated that ART could significantly reduce the viability of RCC cells [118]. It also has been reported that ART inhibited the cell growth of sunitinib-resistant RCC cells [119]. Further study showed that the inhibitory effect of ART on sunitinib-resistant RCC cells was reversed by ferroptosis inhibitor ferrostatin-1 [119]. Moreover, ART also induced a sunitinib-resistant RCC cell cycle arrested at the G0/G1 phase [119]. Based on this evidence, ART may inhibit the growth of sunitinib-resistant RCC cells through the regulation of multiple ways, including ferroptosis.

### Ferroptosis provides a new therapeutic strategy for bladder cancer

Currently, chemotherapy is the major treatment approach for advanced bladder cancer [120]. However, resistance to chemotherapy often results in poorer therapeutic efficacy. Therefore, it is necessary to develop new treatment approaches. Recent studies have examined chemodynamic and molecular targeted therapy as ways to fight bladder cancer. CPNPs, a new tumor-targeted conjugated polymer nanoparticles carrying iron, was reported to suppress the bladder cancer cells through ferroptosis, which might be correlated to the targeting of the endothelin-B receptors (EDNRB) via endothelin-3 surface moieties (EDN3-CPNPs) [121]. CPNPs killed approximately 80% of bladder cancer cells under high doses [121]. In addition, little effects of CPNPs were observed in tumor cells when off-targeted by the EDN3-CPNPs [121]. A series of quinazolinyl-arylurea derivatives have been designed by combining the core pharmacophores of sorafenib and gefitinib [122]. It exhibited a stronger capability to induce cell death compared with gemcitabine in the bladder cancer cell line [122]. Further study found that quinazolinyl-arylurea derivatives-induced cell death could be partially attenuated by Fer-1, a specific inhibitor of ferroptosis [122]. Moreover, Fer-1 might lead to a declination of ROS and an elevation of GSH levels [122]. Thus, a series of quinazolinyl-arylurea derivatives may suppress bladder cancer cell growth by triggering ferroptosis through ROS generation and GSH depletion. These findings may present promising anti-bladder cancer agents.

## Future perspectives of ferroptosis in urologic malignancies

Since a closed association between ferroptosis and urologic malignancies, targeting ferroptosis may be a treatment option for cancer patients. Liu et al. [123] demonstrated that ferroptosis related genes, such as AKR1C3, ALOXE3, ATP5MC3, and CARS1, may serve as the promising prognostic biomarkers and potential drug targets in PCa patients. Yang et al. [111] indicate that ferroptosis inducer erastin is a potential target in treating castration‑resistant PCa patients. For kidney cancer, a number of investigators have found that the tumorigenicity and development of ccRCC might be induced by inhibiting ferroptosis. Thus, ferroptosis could be used to predict prognosis and progression of ccRCC and ferroptosis inducer might be a therapeutic strategy for the sufferers [124, 125]. At present, experimental and clinical studies that have reported the relationship between ferroptosis and bladder cancer are relatively sparse. Only a few bioinformatics analyses [104–108] suggested that ferroptosis might play a role as a molecular biomarker and therapeutic target for bladder cancer. Based on the above evidence, additional well-designed studies are required to explore the underlying molecular mechanisms and clinical outcomes of ferroptosis-mediated therapies for patients with urologic malignancies.

## Conclusions

**Fig. 5:**
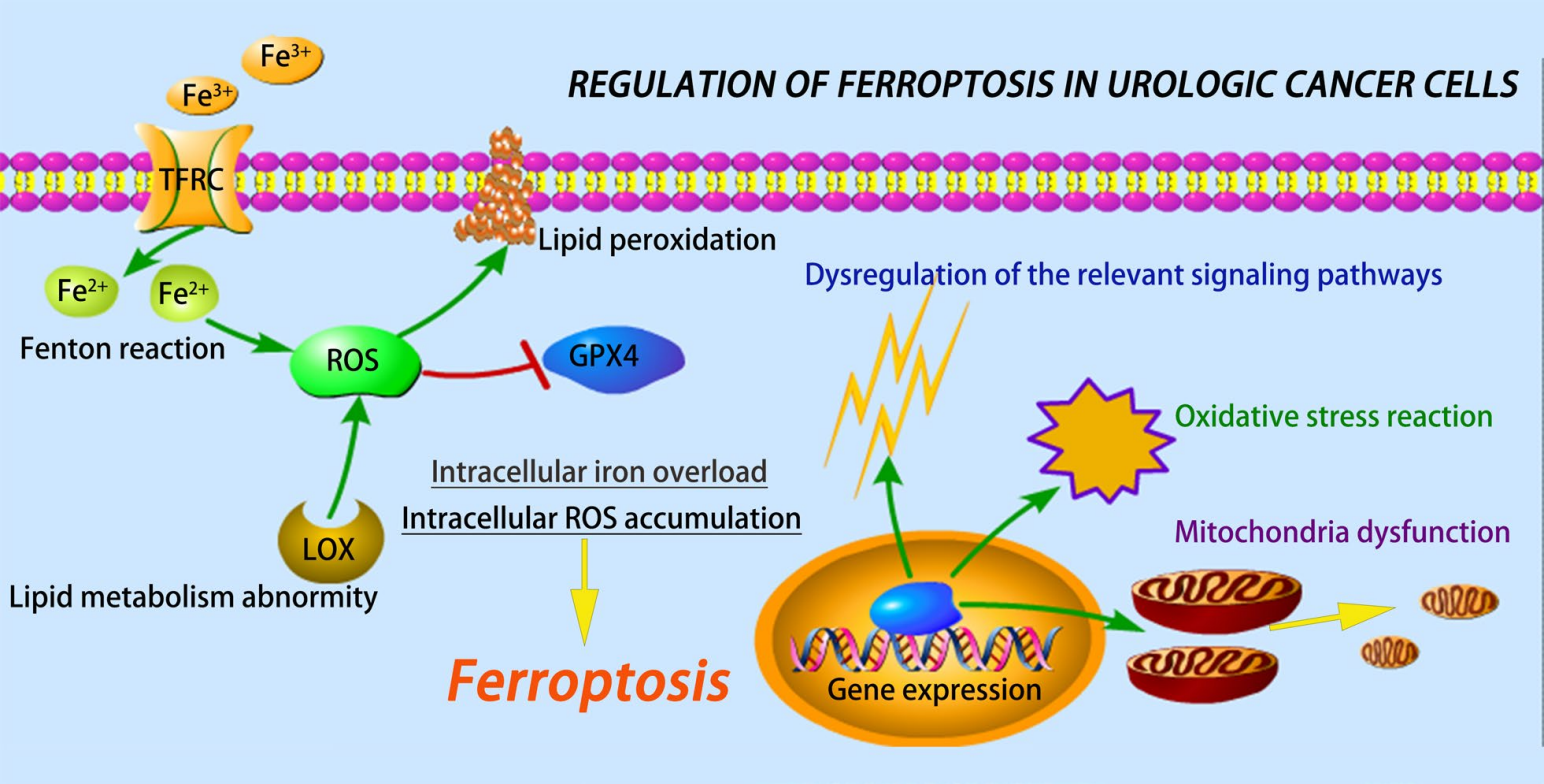
Underlying mechanism of the regulation of ferroptosis in urological cancer cells. Transferrin with Fe^3+^ combines with TFRC enters the cell by endocytosis. Then, ferrous iron (Fe^2+^) is released into the cytoplasm through the Fenton reaction. Iron uptake, along with lipid metabolism abnormity, increases the ROS production, causing lipid peroxidation. GPX4 is a negative regulator of ROS. Intracellular iron overload and ROS accumulation induce ferroptosis. Ferroptosis leads to the aberrant expression of the related genes, resulting in dysregulation of the relevant signaling pathways, oxidative stress reaction, and mitochondria dysfunction. *ROS* reactive oxygen species, *LOX* lipoxygenase, *GPX4* glutathione peroxidase 4, *TFRC* transferrin receptor

In summary, ferroptosis, a novel form of cell death, plays a crucial role in the occurrence, development, progression, and treatment of urological cancers. Table 1 displays the underlying mechanisms of ferroptosis in urological cancers. Figure 5 shows the regulation of ferroptosis in urological cancer cells. Various molecules and signaling pathways are involved in ferroptosis-related urological cancers, including DECR1, PANX2, HSPB1, ACOT8, SUV39H1, NCOA4, PI3K-AKT-mTOR signaling, VHL/HIF-2α pathway, and Hippo/TAZ signaling pathway. Ferroptosis inducers, i.e., erastin and ART, can enhance the anticancer effects of other anticancer drugs in both PCa and kidney cancer. Furthermore, it is speculated that CPNPs and quinazolinyl-arylurea derivatives induce cell death and exert therapeutic effects in bladder cancer via the involvement of ferroptosis. As a result, the elucidation of the molecular mechanism of ferroptosis may provide a novel therapeutic target for urological cancers.

## Data Availability

Not applicable.
